# Factors associated with symptomatic pseudotumors following metal-on-metal total hip arthroplasty

**DOI:** 10.1186/s12891-016-1317-z

**Published:** 2016-11-07

**Authors:** Masahiro Hasegawa, Yohei Naito, Toshio Yamaguchi, Shinichi Miyazaki, Hiroki Wakabayashi, Akihiro Sudo

**Affiliations:** Department of Orthopaedic Surgery, Mie University Graduate School of Medicine, 2-174 Edobashi, Tsu City, Mie 514-8507 Japan

**Keywords:** Total hip arthroplasty, Metal-on-metal, Pseudotumor, Magnetic resonance imaging

## Abstract

**Background:**

Pseudotumors associated with metal-on-metal hips can be symptomatic or asymptomatic. The purpose of this study was to identify the characteristics of pseudotumors associated with pain.

**Methods:**

A total of 239 large-diameter, metal-on-metal total hip arthroplasties (THAs) were performed in 222 patients. Screening for pseudotumors was performed using magnetic resonance imaging (MRI) in all patients who underwent metal-on-metal THA, and 57 patients with 62 affected hips showed pseudotumors. There were 45 women with 49 hips and 12 men with 13 hips affected, with a mean age of 64 years and a mean body mass index (BMI) of 23.9 kg/m^2^. Sixteen hips had symptomatic pseudotumors with pain, and 46 hips were asymptomatic. Pseudotumor size was determined. The anatomical position of pseudotumors was divided into anterior position and posterolateral position. Types of pseudotumors were divided into two types: cystic type; and mixed solid cystic and solid type without a cystic component. The follow-up study of pseudotumors was determined using MRI in 33 patients. The serum cobalt and chromium ion levels were measured in 38 patients after unilateral THA. Univariate and multivariate analyses were performed comparing symptomatic and asymptomatic patients to identify the characteristics of symptomatic pseudotumors.

**Results:**

The mean BMI was 25.4 kg/m^2^ in symptomatic patients and 23.4 kg/m^2^ in asymptomatic patients; a higher BMI was associated with symptoms (*P* = 0.036). Symptomatic pseudotumors were significantly larger (three-fold) than asymptomatic pseudotumors (1812 mm^2^ vs 642 mm^2^, *P* = 0.003). Pseudotumors located in the anterior position were associated with symptoms (*P* = 0.032), and mixed solid cystic and solid type pseudotumors were associated with symptoms (*P* = 0.007). A multivariate analysis showed significant differences only in size (*R* ^2^ = 0.298, *P* = 0.031). No asymptomatic patients with pseudotumors became symptomatic during the follow-up period of MRI evaluation.

**Conclusion:**

Larger size was a significant factor for pain on multivariate analysis.

## Background

Pseudotumors associated with metal-on-metal hips are common in resurfacing and total hip arthroplasty (THA). Pseudotumors, including asymptomatic cases, have been reported in up to 69 % of cases when patients are screened after metal-on-metal hip resurfacing or THA [1–9]. Pseudotumors can be asymptomatic or have a wide variation in presentations, not only with pain and discomfort in the groin, but also with cup loosening or nerve palsy [2, 4, 5, 7, 8]. In addition, the prevalence of pseudotumors was reported to be similar in well-functioning patients and patients with painful metal-on-metal hip implants [10]. Magnetic resonance imaging (MRI) provides sensitive screening of pseudotumors following metal-on-metal hip implants. MRI is ideally suited for assessment of these patients and complements standard clinical evaluation [11, 12].

It remains unclear whether asymptomatic patients with pseudotumors become symptomatic and whether the presence of symptoms correlates with MRI findings and patients’ characteristics. The purpose of this study was to identify the factors associated with symptomatic and asymptomatic pseudotumors. We hypothesized that the pseudotumors would be larger in symptomatic patients and asymptomatic pseudotumors has specific factors not to become symptomatic.

## Methods

A total of 239 large-diameter, metal-on-metal THAs were performed in 222 patients. THA devices included 108 Cormet (Corin, Cirencester, UK), 80 Ultamet (DePuy, Warsaw, IN), and 51 Conserve Plus (Wright Medical Technology, Arlington, TN). Screening for pseudotumors was performed using MRI after THA. Pseudotumors were defined as any mass around the hip joint. MRI studies were conducted in all subjects regardless of symptoms to ensure that asymptomatic pseudotumors could be detected. The mean time at last follow-up with MRI was 49 months (22–85 months). A 1.5-T scanner was used with T1-weighted spin-echo (SE), T2-weighted SE, and short tau inversion recovery (STIR) sequences (Signa; General Electric Healthcare, Waukesha, WI, USA) (Table 1). An eight-channel cardiac coil (GE Healthcare) was used. Two-dimensional fast spin echo images were obtained using modifications to the pulse parameters to reduce susceptibility artifact. A wider receiver bandwidth and oversampling in the frequency encoding axis were used to increase the strength of the readout gradient [13].

**Table 1:** Pulse sequence protocol for MR imaging

	Coronal T1 SE	Coronal T2 SE	Coronal STIR	Axial T1 SE	Axial STIR
Repetition time (msec)	450–560	3000–4090	5000–7600	450–500	5000–7600
Echo time (msec)	9–14	94–100	69–77	13–14	69–77
Inversion time (msec)			150		150
Receiver band-width (Hz per pixel)	300–400	300–400	300–400	300–400	300–400
Flip angle (degrees)	90	90	90	90	90
Field of view (mm)	340 × 340	340 × 340	340 × 340	340 × 340	340 × 340
Matrix	512 × 512	512 × 512	512 × 512	512 × 512	512 × 512
Section thickness (mm)	4–6	4–6	4–6	4–6	4–6

Fifty-seven patients with 62 affected hips showed pseudotumors (28 Cormet, 19 Ultamet, and 15 Conserve Plus). There were 45 women with 49 hips and 12 men with 13 hips, with a mean age of 64 years and a mean body mass index (BMI) of 23.9 kg/m^2^. The primary diagnoses were osteoarthritis in 54 patients and rheumatoid arthritis in three patients. Sixteen hips had symptomatic pseudotumors with pain, and 46 hips were asymptomatic. Fourteen of 16 symptomatic hips had undergone revision surgery. Symptoms disappeared after revision in all revised patients. The Cormet cup was made of cobalt-chromium alloy with a titanium porous coating for bone ingrowth. The Pinnacle cup, which was made of titanium alloy, was a modular cup with a titanium porous coating; a cobalt-chromium alloy liner was inserted in the cup. The Conserve Plus cup was made of a cobalt-chromium alloy with a cobalt-chromium alloy bead coating. The head was made of cobalt-chromium alloy in all implants. The mean head diameter of Cormet devices was 43 mm (40–48 mm). The head diameter for Pinnacle devices was 36 mm in all hips. The mean head diameter of Conserve Plus devices was 47 mm (44–52 mm). The acetabular component inclination angle was measured on anteroposterior pelvic radiographs. The inclination angle was defined as the angle between the line joining the inferior teardrop points and the axis of the opening of the acetabular component. Acetabular anteversion was measured with computer software (Advanced CasePlan Digital Templating Planning Software, Stryker Orthopedics, Mahwah, NJ) [14]. The mean inclination angle of the cup was 44° (23–70°), and the mean anteversion angle was 14° (4–25°).

**Fig. 1:**
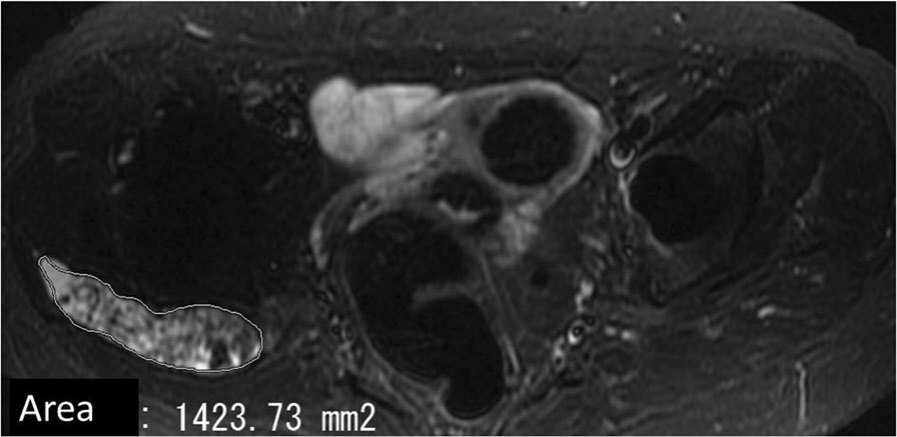
Measurement of pseudotumor size on a magnetic resonance image by manually outlining the greatest size of the mass using computer software

**Fig. 2:**
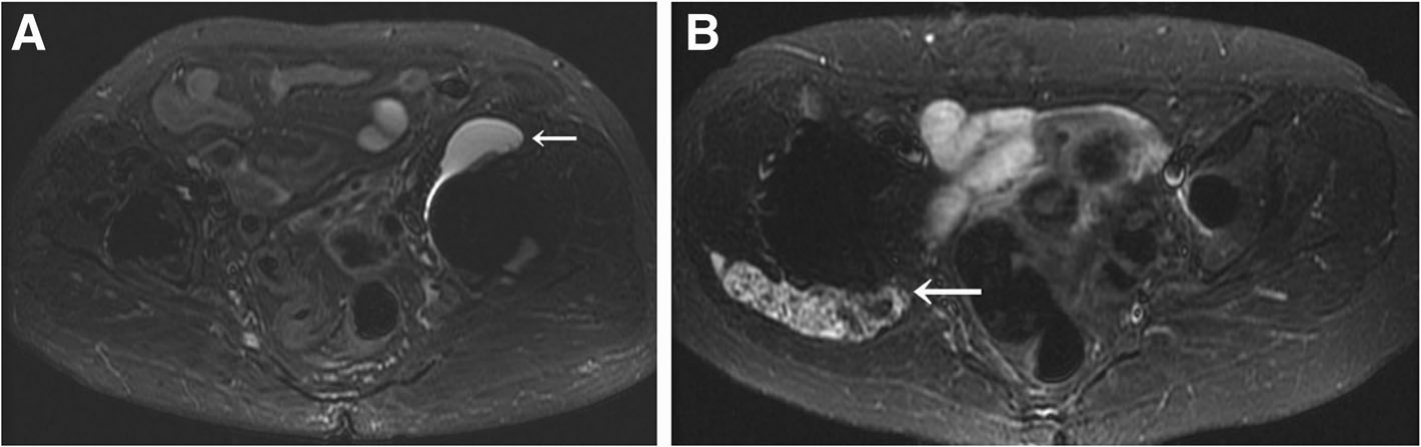
Magnetic resonance images (transverse short tau inversion recovery images) showing pseudotumors (*arrows*) of the cystic type (**a**) and the mixed solid cystic type (**b**)

Pseudotumor size was determined on MRI by one investigator (MH), manually outlining the greatest axial size of the mass. This investigator had 4 years of experience in MRI evaluation of postoperative hips. The area of the pseudotumor was measured using computer software (EV Insite Version 2.10.7.108; PSP Corporation, Tokyo, Japan) (Fig. 1). The scan areas were obtained from the same image for each longitudinal comparison. The anatomical position of pseudotumors was divided into anterior position and posterolateral position. When both positions were involved, the hip was classified based on the larger size. Pseudotumors were located in the anterior position in 23 hips and in the posterolateral position in 39 hips. Types of pseudotumors were divided into two types [4, 15]: cystic type (35 hips) and mixed solid cystic type and solid type without a cystic component (27 hips) (Fig. 2). The follow-up study of pseudotumors was determined using MRI in 33 patients. The mean time between the first MRI and the follow-up MRI was 24 months (8–66 months). Whether asymptomatic patients with pseudotumors became symptomatic was examined, and the growth rate of pseudotumors was determined from follow-up MRIs. The serum cobalt and chromium ion levels were measured closest to the date of the MRI in 38 patients after unilateral THA (11 symptomatic and 27 asymptomatic patients). Cobalt levels were assayed using Inductively Coupled Plasma Mass Spectrometry (Perkin-Elmer SCIEX Elan 6100 DRC ICPMS system; Perkin-Elmer Instruments, Norwalk, CT) at Mayo Medical Laboratories (Rochester, MN), and chromium levels were assayed using a graphite furnace atomic absorption spectrometer (Z-5700; Hitachi Ltd., Tokyo, Japan) with polarization-Zeeman absorption at Mitsubishi Chemistry Medience Co., Ltd. (Tokyo, Japan). Detection limits for each ion were 0.2 μg/L [16]. This study was approved by the local institutional review board, and all patients provided their informed consent.

### Statistical analysis

The Mann-Whitney U test was used to compare age, BMI, femoral head diameter, acetabular cup inclination and anteversion angles, time of MRI screening, pseudotumor size, and serum cobalt and chromium ion levels between symptomatic and asymptomatic patients. The chi-squared test and Fisher’s exact test were used to compare sex, position of pseudotumors, pseudotumor type, and changes of pseudotumor size between the groups. Multiple regression analysis was also performed. Statistical significance was set at *P* < 0.05. Statistical analysis was performed using SPSS version 22 (SPSS, Inc, Chicago, IL).

## Results

The mean BMI was 25.4 kg/m^2^ in symptomatic patients and 23.4 kg/m^2^ in asymptomatic patients; higher BMI was associated with symptoms (*P* = 0.036, Table 2). Symptomatic pseudotumors were significantly larger (three-fold) than asymptomatic pseudotumors (*P* = 0.003, Table 2). Pseudotumors located in the anterior position were associated with symptoms (*P* = 0.032, Table 2), as were mixed solid cystic and solid type pseudotumors (*P* = 0.007, Table 2). Other factors were not significantly different between the groups (Table 2). The growth rate of asymptomatic pseudotumors was 132 ± 502 mm^2^/year. On multivariate analysis, a significant difference was seen only in size (*R* ^2^ = 0.298, *P* = 0.031). BMI (*P* = 0.100), position (*P* = 0.116), and type (*P* = 0.161) showed no significant differences on multivariate analysis. No asymptomatic patients with pseudotumors became symptomatic during the MRI follow-up period.

**Table 2:** Risk factors associated with symptomatic pseudotumors

	Symptomatic	Asymptomatic	*P*
Sex			
Female	15	34	0.154
Male	1	12	
Age (years)^a^	63.1	64.1	0.803
BMI (kg/m^2^)^a^	25.4	23.4	0.036
Head diameter (mm)^a^	42.6	41.7	0.454
Cup inclination (°)^a^	45.2	43.5	0.790
Cup anteversion (°)^a^	14.1	13.3	0.494
Time of MRI screening (months)^a^	45.7	49.7	0.359
Pseudotumor size (mm^2^)^a^	1812	642	0.003
Position of pseudotumor			
Anterior	10	13	0.019
Posterolateral	6	33	
Pseudotumor type			
Cystic	4	31	0.007
Mixed cystic solid and solid	12	15	
Change of pseudotumor size			
Decreased	2	6	0.083
Increased	4	8	
No change	0	13	
Cobalt level (μg/L)^a^	11.7	8.8	0.176
Chromium level (μg/L)^a^	10.3	6.2	0.171

^a^mean

## Discussion

Pseudotumors following metal-on-metal hip arthroplasty are an important problem. Early and accurate diagnosis of a pseudotumor is crucial to plan further management of this potentially devastating complication because late revision surgeries can have poor outcomes [17, 18]. However, previous longitudinal studies demonstrated disappearance of pseudotumors [19–21]. We confirmed that pseudotumors frequently change in size [22] on longitudinal assessment with MRI after metal-on-metal THA. Some patients with pseudotumors following metal-on-metal arthroplasty have severe soft tissue damage resulting in early failure. In contrast, some patients with pseudotumors have no symptoms. Asymptomatic pseudotumors have been reported to occur in 27 to 73 % of cases (Table 3) [2, 7, 8, 10]. It is desirable to determine the characteristics of symptomatic pseudotumors.

**Table 3:** Prevalence of symptoms with pseudotumors and associated factors

Authors	*n*	Symptomatic (%)	Asymptomatic (%)	Factor associated with symptoms
Hart et al. [10]	34	17 (50)	17 (50)	
Sutphen et al. [8]	70	31 (44)	39 (56)	Elevated serum cobalt level
Bisschop et al. [2]	40	11 (28)	29 (73)	Larger pseudotumors
Nawabi et al. [7]	55	40 (73)	15 (27)	Larger pseudotumors
Current study	62	16 (26)	46 (74)	Larger pseudotumors

Chang et al. [3, 23] reported that pseudotumor size was not associated with symptoms. However, other studies [2, 7] demonstrated larger pseudotumors in symptomatic patients, similar to the present study’s finding. Hart et al. [10] confirmed that the presence of a fluid-filled lesion (cystic type) visible on MRI had less clinical importance, but they were concerned about solid pseudotumors. Hauptfleisch et al. [24] reported that predominantly solid (mixed type) pseudotumors were associated with a higher likelihood of symptoms than cystic pseudotumors. The present results support these findings. In terms of metal ion concentrations, a recent study by Sutphen et al. [8] demonstrated that elevated serum cobalt levels might be associated with symptoms. However, the present study, as well as the study by Chang et al. [23], showed no significant correlation with symptoms.

Guidelines recommending follow-up of asymptomatic patients undergoing metal-on-metal hip arthroplasty have already been published [25]; however, the indications for operative revision have yet to be established. In our institution, revision is recommended for patients with painful hips with pseudotumors.

Pseudotumors observed in asymptomatic patients could eventually become symptomatic [23]. One of the strengths of this study was that the follow-up study of asymptomatic patients with pseudotumors was observed, and no asymptomatic patients with pseudotumors became symptomatic during the MRI follow-up period.

This study has some limitations. First, a small number of patients was studied. Second, the follow-up period was short. A longer follow-up study will be necessary. Third, there is no standardized definition of pseudotumor in the literature. It is likely that, in some patients with cystic pseudotumor, its presence is not a result of an abnormal tissue reaction, but rather fluid accumulation [22].

## Conclusions

Higher BMI, larger pseudotumor size, pseudotumors in the anterior position, and mixed solid cystic and solid type of pseudotumors were predictors of symptoms on univariate analyses. For these patients, longer follow-up studies are required. However, larger size was the only significant factor for symptoms on multivariate analysis, and our first hypothesis was verified. If asymptomatic patients with pseudotumors are asymptomatic at follow-up MRI, especially with pseudotumors small in size in the posterolateral position, and of cystic type, further MRI follow-up may not be indicated because these patients have less chance to become symptomatic. Asymptomatic pseudotumors with small in size in the posterolateral position, and of cystic type could be specific factors not to become symptomatic. And our second hypothesis was verified.

## Abbreviations

BMI: Body mass index
MRI: Magnetic resonance imaging
STIR: Short tau inversion recovery
THA: Total hip arthroplasty
